# Ayahuasca and Its Main Component *N,N*-Dimethyltryptamine (DMT) for the Treatment of Mental Disorders: Mechanisms of Action, Clinical Studies, and Tools to Explore the Human Mind

**DOI:** 10.3390/biomedicines14030506

**Published:** 2026-02-25

**Authors:** Alice Melani, Giorgia Papini, Marco Bonaso, Letizia Biso, Shivakumar Kolachalam, Nicola Luigi Bragazzi, Ciro Conversano, Graziella Orrù, Biancamaria Longoni, Marco Scarselli

**Affiliations:** 1 Department of Biology, University of Pisa, 56126 Pisa, Italy; a.melani7@studenti.unipi.it; 2 BIO@SNS Lab, Scuola Normale Superiore, 56126 Pisa, Italy; 3 Department of Surgical, Medical and Molecular Pathology, Critical and Care Medicine, University of Pisa, 56126 Pisa, Italy; giorgia.papini@med.unipi.it (G.P.); ciro.conversano@unipi.it (C.C.); graziella.orru@unipi.it (G.O.); 4 Department of Translational Research and New Technologies in Medicine and Surgery, University of Pisa, 56126 Pisa, Italy; bonasomarco@gmail.com (M.B.); l.biso@studenti.unipi.it (L.B.); kolachalam@outlook.com (S.K.); 5 Aseptic Pharmacy, Charing Cross Hospital, Imperial College Healthcare NHS Trust, London W6 8RF, UK; 6 Department of Clinical Pharmacy, Saarland University, 66123 Saarbrücken, Germany; nicola.bragazzi@uni-saarland.de; 7 Human Nutrition Unit (HNU), Department of Food and Drugs, University of Parma, 43125 Parma, Italy

**Keywords:** psychedelics, ayahuasca, DMT, depression, cognition, consciousness

## Abstract

In recent years, psychopharmacology has experienced a significant challenge, highlighting a renewed and strong scientific interest in psychedelics as breakthrough therapies for mental disorders. Psychedelics can influence cognitive and emotional processes, showing solid therapeutic potential, particularly in treatment-resistant psychiatric disorders. Amongst the most promising compounds, ayahuasca and its main psychoactive component, *N,N*-dimethyltryptamine (DMT), have received considerable attention. Ayahuasca is a psychoactive brew traditionally prepared from the liana *Banisteriopsis caapi* and the leaves of *Psychotria viridis*. Its psychoactive properties derive mainly from DMT, while β-carbolines, which act as monoamine oxidase-A (MAO-A) inhibitors, prevent the metabolic degradation of DMT, enhancing its bioavailability and allowing oral administration. In contrast, in monotherapy, DMT or its analog 5-methoxy-*N,N*-dimethyltryptamine (5-MeO-DMT) is generally administered via alternative routes, like inhalation, intranasal, or intravenous delivery. DMT is primarily a serotonin (5-HT)2A receptor partial agonist, whereas 5-MeO-DMT has a higher affinity for the 5-HT1A receptor compared to 5-HT2A, though other receptor targets are engaged, fostering neuroplasticity and a reorganization of brain networks involved in perception, cognition, and mood regulation. Despite limited clinical trials, current evidence offers an optimistic outlook on DMT and 5-MeO-DMT efficacy for treatment-resistant depression (TRD) and major depressive disorder (MDD), whereas evidence for other mental disorders studies is still preliminary. There are four phase II studies with 5-MeO-DMT and one with DMT for TRD, while there are two phase II studies with DMT fumarate for MDD. Beyond their therapeutic potential, psychedelics also represent powerful tools for exploring the human mind, offering valuable insights into brain function and mental health.

## 1. Introduction

In recent years, the search for new therapeutic strategies for psychiatric disorders has stimulated renewed attention to compounds capable of modulating brain functions through mechanisms distinct from those of conventional treatments. Scientific interest has progressively grown around new compounds to address complex psychiatric disorders that are often resistant to standard pharmacological therapies. In particular, psychedelic substances have emerged as promising candidates due to their effects on brain circuitry involved in emotional regulation, perception, cognition, and mood [1,2]. This renewed interest, commonly referred to as the “psychedelic renaissance,” has been fueled by encouraging clinical results with compounds such as 3,4-methylenedioxymethamphetamine (MDMA), psilocybin, lysergic acid diethylamide (LSD), and the main psychoactive component of ayahuasca, *N,N*-dimethyltryptamine (DMT), which have reached advanced clinical trials for several mental health conditions, including post-traumatic stress disorder (PTSD), treatment-resistant depression (TRD), major depressive disorder (MDD), and generalized anxiety disorder (GAD) [3]. These breakthrough therapies have opened new avenues for treating a range of mental psychopathological conditions, in the so-called psychedelic-assisted psychotherapy [4]. Additionally, psychedelics can induce changes in consciousness, enhance emotional processing, increase introspection and empathy, and reduce negative bias and social withdrawal [5]. Regarding the neurobiological mechanisms responsible for their clinical effects, brain neuroplasticity and changes in neural circuits seem to be essential. On this topic, psychedelics have been shown to profoundly alter several networks such as the default mode network (DMN), the executive control network (ECN), and the salience network (SN), as exemplified by disruptions in cortico-striatal-thalamo-cortical (CSTC) signaling and the relaxation of high-level priors, formalized within the CSTC framework, the relaxed beliefs under psychedelics (REBUS) model, and the revised beliefs after psychedelics (REBAS) model [6,7,8,9].

Furthermore, psychedelics enhance functional connectivity between sensory cerebral areas, decrease the connections between associative brain areas, and reduce the thalamic filtering, thus increasing the sensory processing and rendering the brain more adaptable to external changes.

Among the diverse psychedelic substances, this review investigates ayahuasca and its main psychoactive component DMT, considering their fascinating historical background and widespread utilization, particularly in South America. Ayahuasca, a psychoactive drink of Amazonian origin, has been used for centuries in ritual contexts by various indigenous populations for spiritual and therapeutic purposes [5,10]. Its traditional preparation combines the liana *Banisteriopsis caapi*, which contains β-carboline inhibitors of monoamine oxidase type A (MAO-A), and the leaves of *Psychotria viridis*, which provide DMT, the principal psychedelic compound responsible for hallucinogenic effects. DMT acts primarily as a serotonin (5-HT) 2A receptor partial agonist, inducing neuroplasticity and reorganization of brain networks involved in perception, cognition, and mood regulation. However, DMT is also a partial agonist at 5-HT1A and 5-HT2C receptors. Another similar compound that has received particular attention is the DMT analog 5-methoxy-*N,N*-dimethyltryptamine (5-MeO-DMT), which has a higher affinity for the 5-HT1A receptor compared to 5-HT2A. However, other receptor targets are also involved in the mechanism of action of these two compounds, including σ1 and other 5-HTRs [11]. Despite limited human clinical trials, current evidence offers an optimistic outlook on the efficacy of DMT and 5-MeO-DMT for TRD and MDD, whereas studies are still in progress for the anxiety disorders [4,12,13].

In addition to their use in several mental disorders, psychedelics offer new insights into several aspects of the human mind, including consciousness, near-death experiences (NDEs), and its potential to recover. This review offers a general view of ayahuasca, DMT, and 5-MeO-DMT by analyzing their pharmacokinetic, pharmacodynamic, and clinical properties, including their effects on conscious states.

## 2. Ayahuasca: Composition, Preparation, Pharmacokinetics, and Pharmacodynamics

**Figure 1:**
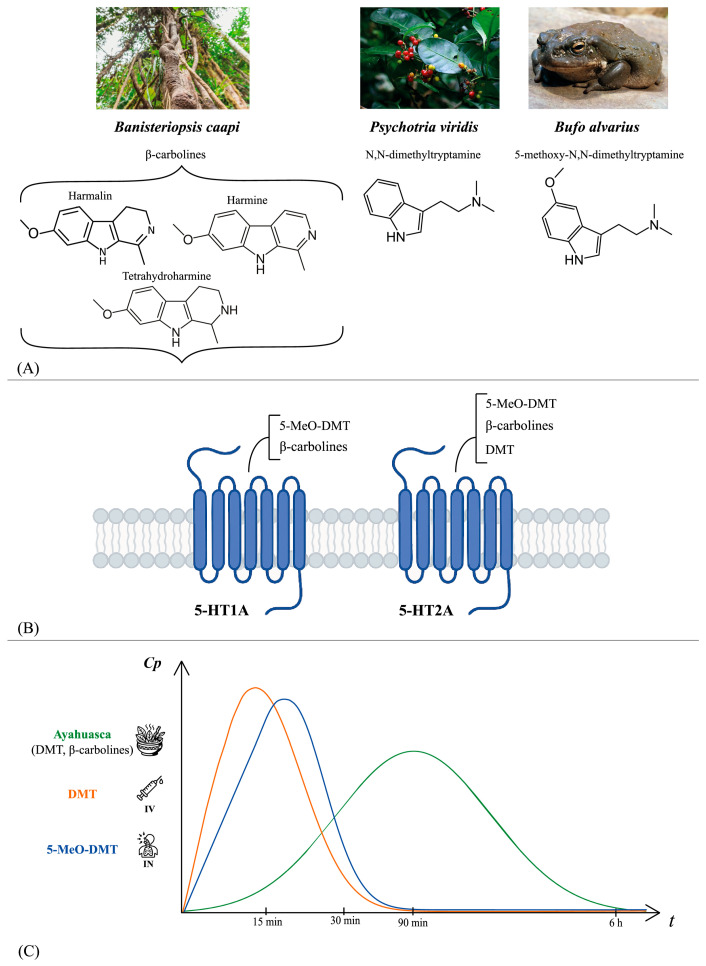
(**A**) Chemical structures of the psychoactive compounds β-carbolines (harmine, harmaline, and tetrahydroharmaline) from *B. caapi* liana, *N,N*-dimethyltryptamine (DMT) from *P. viridis* leaves, and 5-methoxy-*N,N*-dimethyltryptamine (5-MeO-DMT) from *Bufo alvarius* secretions. (**B**) Main 5-HT receptor targets involved in the pharmacological effects of ayahuasca and related tryptamines (DMT and 5-MeO-DMT). β-carbolines interact with 5-HT1A and 5-HT2A receptors and inhibit monoamine oxidase A (MAO-A). 5-MeO-DMT shows a higher affinity for the 5-HT1A receptor than for 5-HT2A, whereas DMT primarily acts as a partial agonist at the 5-HT2A receptor. DMT is also a partial agonist at 5-HT1A and 5-HT2C receptors. (**C**) Schematic curves of plasma concentration (Cp) versus time (t) of DMT derived from ayahuasca orally administered (green), DMT (orange; Intravenous as DMT fumarate (IV)), and 5-MeO-DMT (blue; Inhaled (IN)). Orally administered DMT from ayahuasca shows a delayed peak and prolonged duration, attributable to β-carboline-mediated MAO-A inhibition. Intravenous DMT or inhaled 5-MeO-DMT produces a rapid onset and a shorter duration.

Ayahuasca, also referred to as the “liana of souls” or “the vine of death”, is a hallucinogenic drink that has been used by indigenous tribes in the Amazon region for both ritualistic and therapeutic purposes [5,10,14]. Rivier and Lindgren in 1972 identified the chemicals responsible for the hallucinogenic effects of this drink, which is composed of the liana *B. caapi* and the leaves of *P. viridis* (Figure 1).

The former contains β-carbolines (harmine, harmaline, and tetrahydroharmaline), while the latter contains the main psychoactive ingredient DMT. β-carbolines inhibit MAO-A, thereby preventing the degradation of DMT and increasing its bioavailability [6,14,15,16]. In addition to MAO-A inhibition, β-carbolines have been shown to interact with serotonin receptors independently of DMT. This interaction may contribute to the complex psychoactive profile of ayahuasca by affecting the serotonergic system [7,10,14]. Indeed, it has been demonstrated that 5-MeO-DMT and β-carbolines bind to 5-HT2A and 5-HT1A receptors, thus affecting both the dopaminergic and glutamatergic pathways. In contrast, DMT, the primary psychedelic compound responsible for hallucinogenic effects, acts as a partial agonist at the 5-HT2A receptor (Figure 1). These multifaceted interactions are likely to contribute to the unique effects and emerging therapeutic potential of ayahuasca, including its antidepressant and neuroprotective effects [8,17,18].

Ayahuasca is prepared by boiling a mixture of *B. caapi* and *P. viridis* in water, and the composition of this mixture varies depending on the Amazon region and the ceremony [19,20]. The boiling process is essential to break down plant fibers and facilitate the release of active compounds. The duration of boiling is another critical factor, with prolonged heating resulting in increased extraction of active compounds, thereby enhancing the potency of the mixture. This meticulous preparation ensures the bioavailability of the psychoactive components, which, upon ingestion, induce a series of behavioral and cognitive effects. During preparation, practitioners carefully adjust the bitterness and potency through direct tasting [21,22]. Once the optimal concentration is reached, the decoction is cooled down slowly to preserve the chemical stability of the active compounds and then stored in a cool, dark place away from light and heat to avoid degradation of its properties [23]. In certain instances, supplementary ingredients may be added to modulate the desired effect and potency; indeed, numerous indigenous groups have developed intricate variants of the decoction by incorporating other plants [24].

Ayahuasca may be consumed multiple times during ceremonial practices, and the experience is often accompanied by side effects such as vomiting, nausea, and diarrhea [25]. Upon ingestion, the acute effects of ayahuasca typically manifest within 15 to 30 min and reach their maximum intensity after approximately 90 min (Figure 1). Furthermore, depending on the dosage administered, these effects last from 2 to 6 h [26]. The psychological effects of ayahuasca comprise alterations in the perception of time and space, visual and auditory changes, and cognitive shifts [27,28,29]. Ayahuasca induces a modified and transient state of consciousness characterized by introspection, closed-eyes visualizations, and heightened emotions [26,30,31]. Individuals who have used ayahuasca have reported changes in skin sensitivity, such as tingling or sensations of heat or cold, followed by a strong urge to close their eyes. Their visual imagery is often likened to dream experiences, where the individuals remain aware, and the visions fade as soon as their eyes are opened, and the attention shifts to a specific object [32]. Besides its presence in ayahuasca preparations, DMT is also available in a variety of chemical formulations and preparations, which affect its route of administration and efficacy. While synthetic DMT and its analogs allow precise dose standardization and well-characterized pharmacokinetics, ayahuasca is a multi-component preparation in which β-carbolines enable the oral bioavailability of DMT through MAO-A inhibition and may also contribute to the overall pharmacodynamic and experiential profile [8]. In clinical trials, ayahuasca is generally orally administered and titrated on DMT content, while the presence of β-carbolines is variable, and this can influence its clinical efficacy. For this reason, recently an “ayahuasca-inspired” oral formulation combining DMT and harmine, a MAO-A inhibitor, has been developed in experimental contexts [33]. It should be remembered that DMT and 5-MeO-DMT follow specific controlled-substance regulations, whereas traditional ayahuasca preparations may follow different regulatory legislations depending on the jurisdiction of the country [34].

In clinical settings, DMT cannot be administered orally for its extensive first-pass metabolism mediated by MAO-A in the intestinal mucosa and liver. In fact, DMT is generally used in the form of fumarate or hydrochloride salts to enhance its solubility and stability for intravenous (IV) or inhalation administration [35]. From a pharmacokinetic perspective, DMT demonstrates high bioavailability through IV and inhalation routes, reaching a peak plasma concentration within minutes (Figure 1). Recent research using physiologically based pharmacokinetic modeling has further identified hepatic and pulmonary metabolism as key determinants for DMT clearance [36,37,38].

However, due to the rapid metabolism by MAO-A, the elimination half-life is relatively brief, approximately 15 min [39,40,41]. DMT can also be administered by inhalation or smoking, producing effects that are rapid and short-lived, with an average duration between 5 and 15 min [40,41,42].

In addition to DMT, a related compound that has recently attracted increasing clinical interest is its analog 5-MeO-DMT, a natural tryptamine isolated from several South American plants and from the secretion of the toad *Bufo alvarius*, but which is not present in traditional ayahuasca [43,44]. Pharmacologically, 5-MeO-DMT is distinguished by its higher affinity for the 5-HT1A receptor compared to 5-HT2A. This peculiar receptor profile contributes to a subjective experience characterized more by ego dissolution and states of unity, usually devoid of complex visual content [43,44,45]. The 5-MeO-DMT is pharmacologically inactive when administered orally due to rapid metabolism. This compound undergoes *O*-demethylation via the polymorphic cytochrome P450 2D6 (CYP2D6) enzyme, resulting in the formation of the active metabolite bufotenine, while it is primarily inactivated through deamination by MAO-A [46]. 5-MeO-DMT is frequently co-administered with MAO-A inhibitors, such as harmaline, which inhibits its deamination and thereby extends and enhances the exposure to both the parent compound 5-MeO-DMT and bufotenine. For this reason, the formulations under study are based on alternative routes such as inhalation, intranasal, intramuscular, or intravenous injection. These routes ensure a rapid onset (a few minutes) and a short duration of the experience (about 15 to 90 min), offering operational advantages in the clinical setting (Figure 1). Preliminary studies have reported good tolerability and a rapid reduction in depressive symptoms in clinical settings [43,44,45].

## 3. DMT’s Mechanism of Action

Data from preclinical studies strongly support the involvement of 5-HT receptors in the effects elicited by the classic psychedelics, including DMT, but the whole picture is far more complex [3,47]. The 5-HT receptors have been demonstrated to have a pivotal role, in particular the 5-HT2A receptors. However, the 5-HT-mediated action is not sufficient to induce the hallucinogenic effects, since not all 5-HT2A agonists are hallucinogenic. Indeed, although the 5-HT2A receptors are coupled to Gq protein, they also have biased activity towards the β-arrestin-dependent transduction pathway, suggesting the involvement of distinct signaling pathways downstream receptor activation [13,47,48,49]. On this topic, a recent study has shown that the extent of recruitment of the 5-HT2A-Gq pathway, assessed using the magnitude of the mouse head-twitch response (HTR), predicts the psychedelic potential of substances in mice [13]. This is consistent with the fact that some partial agonists of 5-HT2A (e.g., lisuride) are non-psychedelics. On the contrary, DMT primarily activates the Gq signaling but produces a weak β-arrestin2 recruitment, thus showing a preferential biased activity towards the G protein pathway. Besides 5-HT2A biased activation, DMT has been shown to be a partial agonist at 5-HT1A and 5-HT2C receptors. In addition, this compound has affinities for other serotonergic receptor subtypes, including 5-HT1B, 5-HT6, and 5-HT7, thus supporting a multi-receptor pharmacological profile [11,35]. The 5-HT1A receptor is mostly implicated in the regulation of mood and anxiety, while the 5-HT2C receptor is important for modulating the dopaminergic and limbic circuits [35,50]. Receptors such as 5-HT6 and 5-HT7 are implicated in cognition and synaptic plasticity, further contributing to the broader neuropsychological effects of DMT [11]. Consequently, while 5-HT2A plays a pivotal role in mediating the psychedelic effects, the relevance of additional serotonergic receptors is a subject for further research [11].

Using the HTR test and radioligand binding assays, a recent study in rodents was able to further elucidate the DMT mechanism of action, evaluating the interplay between 5-HT2A and mGluR2 metabotropic glutamate receptors [50]. The study effectively demonstrated that MDL100907 (5-HT2A inverse agonist) fully blocked the discriminative stimulus effects of DMT and the HTR, correlating with the hallucinogenic effects of the compound. On the contrary, LY379268 (mGluR2/3 agonist) produced a potent but only partial blockade of the discriminative stimulus effects of DMT. Overall, these data suggest that the 5-HT2A receptor presumably plays a major role in mediating the effects of DMT, while the mGluR2 most likely partially modulates the hallucinogenic effects of the compound.

Apart from interfering with serotonergic and glutamatergic transmission, DMT has been shown to bind to sigma-1 (σ1) receptors. The σ1 receptor acts as an intracellular chaperone between the endoplasmic reticulum (ER) and mitochondria, performing unique functions such as serving as a tunnel for lipid transport and Ca^2+^ signaling (through the IP3 receptor) between the ER and mitochondria [35,51], and activating the antioxidant response elements (AREs) [52]. Thus, as an agonist of σ1 receptor, DMT is able to act as an indirect antioxidant factor. It is interesting to note that the σ1 receptors are widely distributed throughout the body, including the central nervous system (CNS), and have been linked to various neurobiological disorders and conditions, including Alzheimer’s disease, Parkinson’s disease, cancer, and major depression [53,54].

## 4. DMT, Neural Circuitries, and Brain Entropy

**Figure 2:**
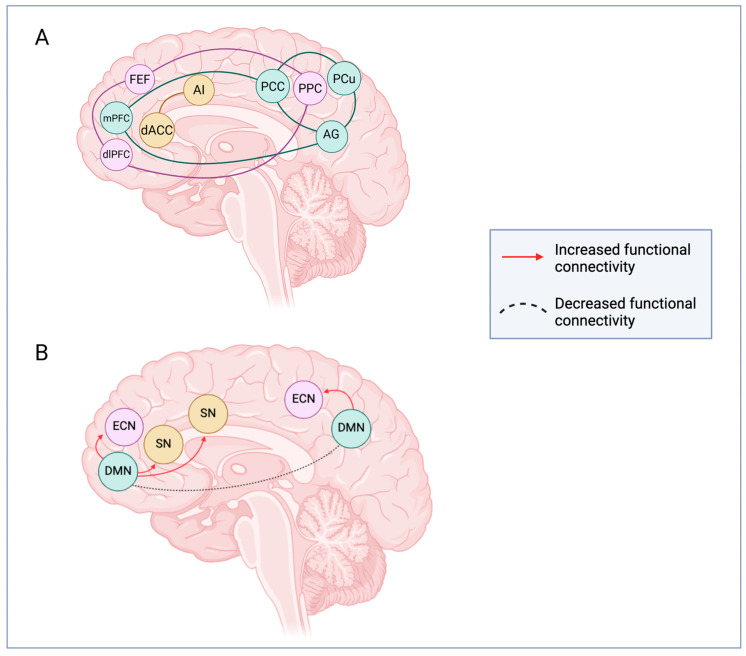
Effects of psychedelics on brain connectivity among the DMN, ECN, and SN networks. (**A**) Main regions recruited in the DMN (green), ECN (purple), and SN (orange) networks. (**B**) Psychedelics increase connectivity between the DMN and SN, as well as between the DMN and ECN, along with reduced connectivity within the hubs of the DMN. DMN: default mode network; ECN: executive control network; SN: salience network; AG: angular gyrus; AI: anterior insula; dACC: dorsal anterior cingulate cortex; dlPFC: dorsolateral prefrontal cortex; FEF: frontal eye field; MPFC: medial prefrontal cortex; PCu: precuneus; PCC: posterior cingulate cortex; PPC: posterior parietal cortex. Created in BioRender. Bragazzi, N. L. (2026) https://BioRender.com/ht8fm40; accessed on 18 February 2026.

Psychedelics have been shown to change several networks, such as DMN, ECN, SN, and the connection between them. For instance, they reduce connectivity within the DMN brain regions, but they increase connections between DMN and ECN and between DMN and SN, contributing to cognitive restructuring, cognitive flexibility, and salience to external/internal stimuli (Figure 2).

**Figure 3:**
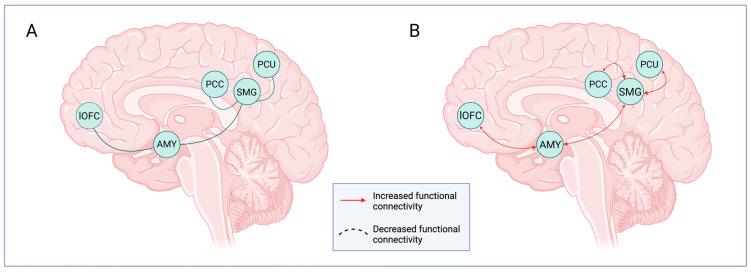
DMT’s effects on “social brain” circuitry. (**A**) Main regions recruited by DMT’s effects: IOFC, involved in reward processing, AMY, and SMG, which are parts of the so-called “social brain”. (**B**) DMT is able to increase the SMG connectivity with the precuneus, the AMY, the IOFC, and the posterior cingulate gyrus. These changes are consistent with the models, which propose that psychedelics increase global functional connectivity and cause the simultaneous disintegration of the associative networks. AMY: amygdala; SMG: supramarginal gyrus; IOFC: orbitofrontal cortex. Created in BioRender. Bragazzi, N. L. (2026) https://BioRender.com/9fe0zso; accessed on 18 February 2026.

In addition, due to its peculiar capability to interfere with social cognition circuits, DMT is considered to be a potential tool not only in mood disorders but also in disorders involving social–emotional deficits, such as autism spectrum disorders (ASDs) [55]. To understand how DMT is able to cause these effects, seed-based connectivity was measured on adult volunteers by analyzing the two core regions of the so-called “social brain”, which are the posterior supramarginal gyrus, within the temporoparietal junction (TPJ), and the amygdala [55]. Connectivity in the supramarginal gyrus is considered to be crucial for the switching between self- and other-related representations, and this self-other distinction seems to be a necessary component of many social, perceptual, motor, and cognitive tasks and functions [56]. The study showed that DMT is able to increase the supramarginal gyrus connectivity with the precuneus, the posterior cingulate gyrus, the amygdala, and the orbitofrontal cortex, which are key limbic and reward brain areas (Figure 3). These data are consistent with recent models of psychedelic action, which propose that psychedelic substances generally increase global functional connectivity while causing the simultaneous disintegration of the associative networks [57]. In addition, a study has found that the administration of DMT leads to increased functional connectivity between the amygdala and the left orbitofrontal cortex, regions involved in emotion processing and the subjective experience of affective value [58]. Thus, DMT is able to modulate brain connectivity in socio-emotional and affective-value circuits, thereby supporting its potential role as a therapeutic agent for the treatment of many psychiatric disorders.

**Figure 4:**
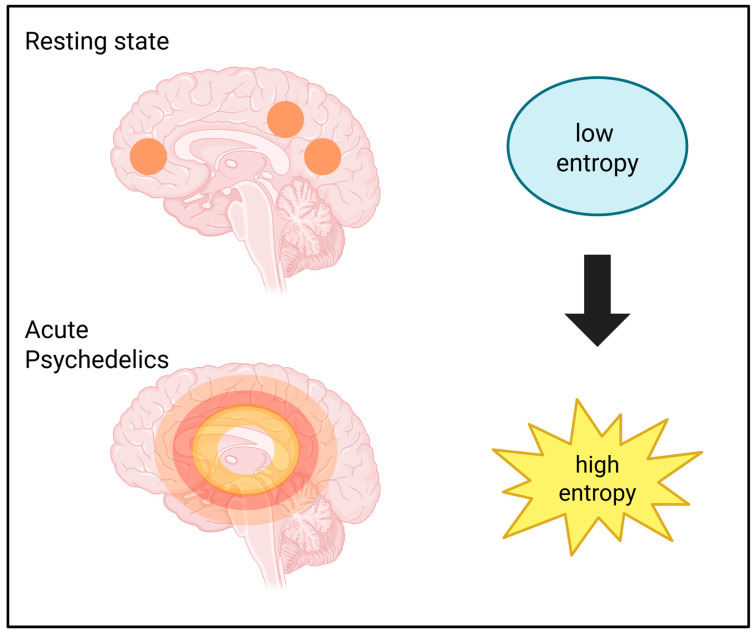
A schematized effect of psychedelics on brain entropy. Created in BioRender. Bragazzi, N. L. (2026) https://BioRender.com/ki6f2zb; accessed on 18 February 2026.

Another common characteristic of psychedelics is the increase in global brain entropy, a hypothesis proposed by Carhart-Harris et al. (2014) [59]. fMRI studies of functional connectivity of human subjects taking the psychoactive beverage ayahuasca have reported an enhancement of the Shannon entropy [60,61]. This is coherent with other findings showing that psilocybin increases entropy within the DMN, together with other findings reporting evidence of increased entropy caused by the lysergic acid diethylamide (LSD) (Figure 4) [62,63,64]. The above evidence correlates with electroencephalogram (EEG) studies in which the effects of IV administration of DMT on spontaneous brain activity were investigated [65].

The subjective effects of IV administration of DMT have a rapid onset and are characterized by vivid visual imagery and somatic effects, resembling a world analog experience (meaning, internal representation of the external world), suggesting that DMT could be a powerful scientific tool to analyze the neurobiology of consciousness.

The study found that the DMT-induced altered state, characterized by the typical visual effects, was accompanied by a marked decrease in total spectral power in the alpha and beta bands, together with an increased signal diversity. Alpha is the most important rhythm of the resting-brain, associated with high-level psychological functions and predictive processing, which, in fact, has been shown to be disrupted under psychedelics. Furthermore, this pattern is coupled with the emergence of delta and theta oscillations, particularly in the medial temporal lobe areas. These altered power spectra can be linked to the so-called “DMT breakthrough experience”, a perceptual mechanism through which the brain switches from the processing of exogenously incoming information to a state in which the processing is endogenously driven, similar to classical rapid eye movement (REM) sleep dreaming [66]. Considering that increased alpha power and decreased delta power have been found in individuals with depression [67], there is substantial evidence to hypothesize that DMT can be used to ameliorate the psychopathology of depression.

## 5. Overview of Major Clinical Trials of Ayahuasca, DMT, and 5-MeO-DMT in Mental Disorders

In recent years, ayahuasca and its main psychoactive compound DMT have attracted growing attention as potential therapeutic agents in several psychiatric disorders. This renewed interest is part of the psychedelic renaissance, supported by rigorous clinical trials on compounds such as psilocybin, MDMA, and LSD, which have shown clinical efficacy and safety for the treatment of PTSD, TRD, MDD, and GAD [3]. While clinical research on ayahuasca and DMT is still at earlier stages, preliminary findings suggest promising antidepressant effects, rapid onset of action, and overall good tolerability. Current trials on ayahuasca and DMT are mainly focused on TRD, MDD, and GAD, with no studies specifically targeting PTSD at the moment. In the following section, we provide a structured overview of the most relevant and advanced clinical trials conducted in humans with ayahuasca, DMT, and its analog 5-MeO-DMT, highlighting their therapeutic and pharmacological characteristics.

### 5.1. Clinical Studies on Ayahuasca, DMT, and 5-MeO-DMT in TRD

The most advanced clinical studies evaluating the therapeutic efficacy of ayahuasca and DMT in TRD are characterized by different methodological approaches and drug formulations. In particular, traditional oral administration of ayahuasca and inhaled formulations of DMT are the most commonly used routes of administration.

Clinical trial NCT02914769 is one of the first randomized, double-blind, placebo-controlled studies to investigate the efficacy and safety of ayahuasca in TRD [68]. This phase 1/2 study enrolled a total of 35 patients, 29 of whom were included in the final analysis. Participants were randomized to receive a single oral dose of ayahuasca containing 0.36 mg/kg DMT (n = 14) or an inactive placebo (n = 15) [12]. Administration was conducted in a controlled clinical setting with preparatory guidance and continuous psychological support throughout the session, although no formal structured psychotherapy was provided. The primary objective was to evaluate the antidepressant efficacy of the substance, as measured by changes in severity of depression on the Hamilton Depression Rating Scale (HAM-D), comparing baseline to seven days after administration. The secondary outcome was the change in Montgomery-Åsberg Depression Rating Scale (MADRS) scores from baseline to 1, 2, and 7 days after administration. Secondary endpoints included the clinical response rate (≥50% reduction in MADRS score) and the clinical remission rate (HAM-D ≤ 7 or MADRS ≤ 10) [12]. Ayahuasca showed a rapid and significant antidepressant effect compared to placebo. On the HAM-D scale, the mean score after 7 days decreased from 24.07 ± 5.34 (baseline) to 9.72 ± 7.39 in the ayahuasca group and from 19.73 ± 4.59 (baseline) to 16.92 ± 7.36 in the placebo group (*p* = 0.019; Cohen’s d = 0.98, a large effect size). On the MADRS, the mean score after 7 days decreased from 36.14 ± 6.12 (baseline) to 11.58 ± 10.27 in the ayahuasca group and from 30.13 ± 5.55 (baseline) to 26.76 ± 10.11 in the placebo group (*p* < 0.0001; Cohen’s d = 1.49, a large effect size). The difference was already significant on the first day (12.65 ± 10.27 vs. 21.49 ± 10.90; *p* = 0.04; Cohen’s d = 0.84, a large effect size) and further widened on the second day (10.32 ± 10.44 vs. 19.09 ± 10.44; *p* = 0.04; Cohen’s d = 0.84, a large effect size). The clinical response was also higher in the ayahuasca group. On the MADRS, the response rate (≥50% reduction in score) was 64% in the ayahuasca group versus 27% in the placebo group at day 7 [Odds Ratio (OR) = 4.95 (95% CI 1.11–21.02); *p* = 0.04; Number Needed to Treat (NNT) = 2.66]. The remission rate (MADRS ≤10) was higher in the ayahuasca group (36% vs. 7%), with a trend toward significance [OR 7.78 (95% CI 0.81–77.48); *p* = 0.054; NNT = 3.44]. On the HAM-D scale, the response rate at day 7 was 57% in the ayahuasca group versus 20% in the placebo group [OR 5.33 (95% CI 1.11–22.58); *p* = 0.04; NNT = 2.69], while the remission rate was 43% versus 13%, respectively [OR 4.87 (95% CI 0.77–26.73); *p* = 0.07; NNT = 3.39]. The treatment was generally well tolerated, and the most common adverse events, such as nausea, vomiting, and sensory alterations, were mild and rapidly resolved with no serious adverse events recorded [12].

A second clinical study, NCT04698603, a randomized, open-label study, explored the efficacy and safety of GH001, an inhaled formulation of 5-MeO-DMT designed to produce rapid, short-lasting effects [69]. The trial enrolled 16 patients with TRD, divided into two distinct phases. In phase 1, 8 patients received a single dose of GH001, equal to 12 mg (n = 4) or 18 mg (n = 4) [4]. The primary objective was to evaluate the safety and tolerability of the compound, using clinical and psychometric measures, including the Brief Psychiatric Rating Scale (BPRS), the Clinician Administered Dissociative States Scale (CADSS), the Columbia-Suicide Severity Rating Scale (C-SSRS), the Psychomotor Vigilance Task (PVT), and the Digit Symbol Substitution Task (DSST). In phase 2, an additional 8 patients were treated with an individualized dosing regimen (IDR), which included up to three cumulative administrations (6 mg, 12 mg, and 18 mg) on the same day, spaced by 3 h apart. Subsequent doses were administered only in the absence of peak psychedelic experience, assessed using the Peak Experience Scale (PES). The primary endpoint of phase 2 was the proportion of patients in depressive remission (MADRS ≤ 10) on day 7. Secondary endpoints, common to both phases, included the change in MADRS score at 2 h, 1 day, and 7 days after administration, as well as the clinical response rate (≥50% reduction in MADRS score). The study did not include structured psychotherapeutic interventions, and interactions were limited to screening and outcome assessments, with preparation and support provided as part of standard medical care [4]. GH001 demonstrated a rapid and marked antidepressant effect, particularly evident in phase 2. The remission rate at day 7 was 50% in the 12 mg group (n = 2/4), 25% in the 18 mg group (n = 1/4), and 87.5% in the IDR group (n = 7/8), with the latter achieving the primary endpoint in a statistically significant manner (*p* < 0.0001; 95% CI = 0.473–0.997). The mean reduction in MADRS score from baseline to day 7 was −21 points (−65%) in the 12 mg group, −12.5 points (−40%) in the 18 mg group, and −24.4 points (−76%) in the IDR group. In 6 of the 10 patients who achieved remission, improvement was visible as early as 2 h after administration. The treatment was generally well tolerated, and no serious adverse events were recorded. The reported events (such as headache, nausea, abnormal sensations, or flashbacks) were mostly mild or moderate and resolved spontaneously. Assessments of dissociation (CADSS), psychiatric symptoms (BPRS), vital signs, and cognitive functions (PVT, DSST) did not reveal clinically significant alterations [4].

The larger and more methodologically rigorous GH001-TRD-201 trial NCT05800860 was a continuation of the GH001 clinical program. This randomized, double-blind, placebo-controlled, phase 2b study included 81 patients with TRD, 40 of whom were treated with GH001 and 41 with placebo. The study consisted of a 7-day double-blind phase followed by a 6-month open-label extension (OLE) phase for eligible participants. GH001 was administered using an IDR, consisting of up to three escalating doses (6, 12, and 18 mg) given on a single day, with subsequent doses administered only when a peak experience was not achieved. The primary objective of this study was to assess the efficacy of a single-day IDR of GH001 versus placebo in reducing depressive symptoms, as measured by MADRS on day 7. Secondary objectives included evaluating the impact of GH001 on other measures of depression, anxiety, and quality of life during both the double-blind and OLE phases, as well as assessing its safety and tolerability [70].

As reported on the trial sponsor’s website, psychotherapeutic intervention was not a component of either the double-blind or the open-label extension phases of this trial. Treatment with GH001 achieved the primary endpoint, resulting in a mean reduction in the MADRS score of −15.2 at day 8 from baseline, compared to a mean increase of +0.3 in the placebo group. The clinical benefit, equal to a placebo-adjusted difference of −15.5 points (*p* < 0.0001), was rapid and significant. All secondary endpoints were also achieved, with statistically significant improvements compared to placebo in the Clinical Global Impressions-Severity scale (CGI-S), Hamilton Anxiety Rating Scale (HAM-A), and Quality of Life Enjoyment and Satisfaction Questionnaire–Short Form (Q-LES-Q-SF). The remission rate (MADRS ≤ 10) in the GH001 group was 57.5% on day 8, compared with 0% in the placebo group (*p* < 0.0001). At six-month follow-up, 77.8% of patients who had completed the OLE phase were still in remission, often after a limited number of doses (1–4 total treatments). Among those who achieved remission as early as day 8, 91.7% maintained their response up to six months. From a safety perspective, GH001 was well tolerated and no serious adverse events or reports of suicidal ideation were reported. All treatment-emergent adverse events were mild or moderate in severity and, in the majority of cases, transient. No clinically significant changes in vital signs, nor post-treatment dissociation or sedation were observed. Almost all patients (97.4%) were ready for discharge within one hour of the last dose [71].

Complementing the clinical studies of 5-MeO-DMT in TRD, so far explored mainly through the GH001 formulation, is the independent development program of BPL-003, an intranasal analog currently in advanced clinical evaluation. Two studies have investigated the efficacy and safety of this molecule: an open-label phase 2a study NCT05660642 [72], and a phase 2b study NCT05870540 [73], approved by the Food and Drug Administration (FDA) as the largest study ever conducted on 5-MeO-DMT in TRD.

The phase 2a study NCT05660642 was conducted in two independent arms, both open-label. According to the trial registry, BPL-003 was administered in combination with psychological support provided before, during, and after dosing. In Arm A, 12 patients with TRD who were not taking concomitant antidepressants received a single intranasal administration of BPL-003 at a dose of 10 mg, as reported in company press releases [72]. Preliminary results indicate that 55% of patients achieved a clinical response (≥50% reduction in MADRS score) as early as day 2, with remission rates (MADRS ≤ 10) of 55% at week 4 and 45% at week 12. The treatment was well tolerated with no serious adverse events reported. In arm B, 12 additional TRD patients on stable treatment with a selective serotonin reuptake inhibitor (SSRI) received the same intranasal dose. Preliminary data released in a company press release show a mean reduction in MADRS scores of 18 points at day 2, 19 points at week 4, and 18 points at week 12 compared to baseline, with a rapid and sustained antidepressant effect over time. Also in this arm, the treatment showed a good tolerability profile with no serious adverse events reported and with discharge within two hours of administration. These results support the compatibility of the administration of BPL-003 with SSRIs, suggesting potential for their use in combined therapeutic contexts, similar to the use of intranasal esketamine.

The phase 2b study NCT05870540 is a multicenter, randomized, double-blind, placebo-controlled study that has so far enrolled 196 patients with TRD. According to the trial registry, BPL-003 is administered in combination with psychological support provided before, during, and after each dosing session. There are five experimental arms with different intranasal dosing strategies (single low, medium, and high doses; monophasic and biphasic modalities), as well as an active comparator (active placebo). The published protocol does not list specific doses, but according to the trial sponsor, the doses under evaluation range from 8 to 12 mg, compared to a sub-perceptual dose. The primary endpoint is the change in the MADRS score at 8 days post-dose. Secondary endpoints include clinical response rate, remission, and safety assessment over 8 weeks of follow-up. Results are not yet available, and top-line data were expected in the second half of 2025.

A recent clinical trial, NCT06094907, conducted between October 2023 and March 2024, represents the first phase 2a clinical study to evaluate the efficacy and safety of free-form DMT, administered by inhalation, in patients with TRD [74]. This randomized, open-label study enrolled 14 patients who were treated with a fixed-dose escalation regimen on the same day: an initial administration of 15 mg, followed by a second dose of 60 mg after 90 min. The protocol incorporated structured psychological preparation prior to dosing and standardized integration sessions immediately after each administration and during follow-up [75]. The primary endpoint was the mean change in MADRS-assessed depression severity from baseline to day 7 after the dosing session. Secondary endpoints included changes in MADRS at day 1, day 14, and month 1; the clinical response rate (≥50% reduction in MADRS score); the remission rate (MADRS ≤ 10); the change in the Patient Health Questionnaire-9 (PHQ-9) from baseline to day 1, 7, 14, and 1 month; and the change in Altered States of Consciousness (ASC) during the acute effects of DMT [75].

The preliminary report included data from only six patients who completed all assessments up to one month. Results showed a rapid and sustained reduction in the severity of depressive symptoms. The mean baseline MADRS score was 32.5 (±2.59), with a mean reduction of −22 points on day 7 and −17 points at four weeks. Changes were statistically significant at all time points: day 1 (*p* < 0.0001; Hedge’s g = 4.70, a very large effect size), day 7 (*p* = 0.0002; Hedge’s g = 2.89, a very large effect size), day 14 (*p* = 0.0002; Hedge’s g = 3.34, a very large effect size), and month 1 (*p* = 0.003; Hedge’s g = 1.80, a very large effect size). The clinical response rate, defined as a ≥ 50% reduction in the MADRS score from baseline, was 83.3% (n = 5/6) at days 1, 7, and 14, and was maintained at 66.7% (n = 4/6) at four weeks. The remission rate (MADRS score ≤ 10) followed a similar pattern: 66.7% (n = 4/6) of patients achieved remission at days 1, 7, and 14, while 50% (3/6) maintained this status at the 1-month follow-up. The PHQ-9 scores also showed a significant reduction parallel to the MADRS score at all time points. Statistical analysis confirmed a significant time effect also for this scale (*p* = 0.006; η_p_^2^ = 0.68, a very large effect size), suggesting a clinically relevant improvement in perceived depressive symptomatology. The treatment was well tolerated with no serious adverse events reported. For both administrations, a temporary but not clinically relevant increase in blood pressure, heart rate, and peripheral oxygen saturation was observed, which resolved spontaneously. Psychedelic experiences were subjectively intense, positive, and resulted in mild effects on total ASC scores [75].

Across the studies reviewed, the integration of structured psychological support varied considerably, ranging from formal preparatory and integration sessions to protocols limited to supportive medical care without structured psychotherapy. As concomitant psychotherapy is commonly considered an important component in psychedelic-assisted treatment models, these differences represent a relevant methodological variable when interpreting clinical outcomes and their durability.

Table 1 summarizes the major clinical trials described in this paragraph.

### 5.2. Clinical Studies on Ayahuasca and DMT in MDD

Among the most advanced and recently completed clinical trials evaluating DMT in MDD, the phase 1/2a study NCT04673383 represents a pivotal step. This two-part study assessed the safety, tolerability, and preliminary efficacy of SPL026, an IV formulation of DMT fumarate, in psychedelic-naïve healthy volunteers (Part A) and in patients with moderate-to-severe MDD (Part B, based on HAM-D scores at baseline) [76]. Before progressing to clinical testing in patients with MDD, SPL026 was evaluated for its safety and tolerability in a phase 1 study (Part A) involving 32 psychedelic-naïve healthy volunteers. Participants received a single IV dose of SPL026 (9, 12, 17, or 21.5 mg) or placebo, infused in two stages over 10 min to gently induce the psychedelic experience and enhance tolerability. All participants received structured psychological support, based on a relational therapeutic model centered on psychological flexibility. This included preparation sessions, support during the experience, and integration sessions post-dose. SPL026 showed a favorable safety and tolerability profile with no serious adverse events and only mild-to-moderate transient side effects (mostly local catheter reactions, headache, or sleep disturbances) [43,77]. Pharmacodynamic analysis showed dose-dependent increases in mystical-type experiences (as measured by the Mystical Experience Questionnaire), ego dissolution, and subjective intensity (as measured by the Intensity Rating Visual Analogue Scale), especially at doses ≥17 mg. The 21.5 mg dose was selected for the subsequent phase 2a trial in MDD, as it provided the most consistent and tolerable psychedelic experience without safety concerns. Despite transient increases in blood pressure post-dose (especially at higher doses), no clinically significant cardiovascular effects were observed and no effects on suicidality were noted across 90 days of follow-up [43,77]. In Part B, the patients received up to two single doses of SPL026, administered two weeks apart with supportive psychotherapy: the first dose was randomized, double-blind, and placebo-controlled, whereas the second dose was open-label and consisted of active SPL026 [76]. SPL026 demonstrated a rapid, robust, and durable antidepressant effect. The primary endpoint was met with a placebo-adjusted reduction in the MADRS score of −7.4 points at two weeks (*p* = 0.02). Rapid improvement was already evident at week 1, with a difference of −10.8 points compared to placebo (*p* = 0.002). Clinically, 50% of the patients achieved remission (MADRS ≤ 10) already in the first week and 39% in the second, and the treatment response rates were 44% and 35%, respectively. Long-term analysis showed a lasting improvement in symptoms in patients who had received at least one active dose; the mean reduction in MADRS reached −15.4 points at 12 weeks, with no clinically relevant differences between those who had received one or two administrations. Among patients in remission in the first 3 months, 64% maintained the state of remission up to the sixth month. Anxiety symptoms assessed using the State-Trait Anxiety Inventory—Trait anxiety (STAI-T) scale also showed a significant improvement: −13.5 points compared to baseline in the first week versus −1.9 in the placebo group, and −11.0 versus −3.4 in the second. The tolerability profile was favorable, and no serious adverse events or episodes of suicidality were reported. AEs were generally mild or moderate and resolved either during or shortly after the session. No clinically relevant changes in vital signs, electrocardiogram (ECG), or laboratory tests were observed [76].

Building upon previous findings, the phase 1b trial NCT05553691 investigated the safety, tolerability, and exploratory efficacy of a single 27.5 mg IV dose of SPL026 administered with psychological support in patients with moderate-to-severe MDD [78]. This open-label study enrolled 17 patients, divided into two cohorts: one composed of individuals on stable SSRI treatment with inadequate symptom relief (SSRI cohort, n = 12), and the other composed of patients not taking any antidepressants at baseline (non-SSRI cohort, n = 5). All participants received a single SPL026 infusion and underwent structured psychotherapy. Efficacy was assessed using the MADRS score, the Beck Depression Inventory (BDI) questionnaire, and the STAI-T scale. As reported on the trial sponsor’s website, the treatment was well tolerated in both cohorts, and no serious adverse events, suicidality, or psychotic symptoms were reported. Most AEs were mild or moderate and transient. In total, 35 adverse events were recorded (27 in the SSRI group, 8 in the non-SSRI group), with no clinically relevant abnormalities in vital signs, ECG, or laboratory parameters [78]. At baseline, the mean MADRS score was 28.8 in the SSRI cohort and 35.4 in the non-SSRI cohort, whereas the mean BDI scores were 30.0 and 36.0, respectively. By week 4, the SSRI group showed a significant reduction in depressive symptoms with a mean MADRS of 3.0 and a mean BDI of 3.1. In the non-SSRI cohort, MADRS decreased to 16.0 and BDI to 14.8. Clinical response (≥50% MADRS reduction) was achieved in 100% of SSRI patients and 80% of non-SSRI patients. Remission (MADRS ≤ 10) was reported in 92% of the SSRI group and 20% of the non-SSRI group. These consistent improvements across both clinician-rated and self-reported outcomes suggest that concomitant SSRI therapy may potentiate the antidepressant effects of SPL026 [78].

Table 2 summarizes the major clinical trials described in this paragraph.

### 5.3. Clinical Studies on Ayahuasca and DMT in GAD

To date, there are no completed clinical trials specifically evaluating ayahuasca or DMT in GAD. However, growing interest in the anxiolytic potential of DMT stems from findings in patients with MDD, where IV administration of SPL026 led not only to robust antidepressant effects but also to significant reductions in anxiety symptoms, as measured by the STAI-T scale. These results support the potential of DMT and its analogs in anxiety-related conditions, especially considering the high rates of comorbidity between depression and GAD [79].

Building on this rationale, a phase 2 trial NCT06051721 is currently underway to evaluate the safety and efficacy of CYB004, a deuterated DMT analog (dDMT) optimized from insights gathered across five prior clinical studies [80]. This randomized, double-blind study is assessing intramuscular (IM) administration of CYB004 combined with EMBARK™, a structured psychotherapy program, in 36 participants with moderate-to-severe GAD (baseline GAD-7 score ≥ 10) [77].

Characteristics of this study are summarized in Table 3.

## 6. Psychedelics as Tools to Explore the Human Mind

Besides their potential in the medical field, psychedelics have been investigated as powerful tools to explore several aspects of the human mind, including consciousness.

Consciousness, the cornerstone of self-awareness and subjective experience, remains one of the greatest enigmas in neuroscience. While theories attempt to conceptualize conscious processing, offering insights into its functional and structural aspects, researchers continue to investigate the neural mechanisms underlying conscious states. Recent evidence highlights the pivotal role of Layer 5 (L5) pyramidal neurons, known for their integrative capacity across cortical and thalamic circuits, in regulating consciousness [81]. Complementing these findings, psychedelics have emerged as effective tools for exploring altered states of consciousness, thus providing novel avenues to study the boundaries between wakefulness, dreaming, and disrupted conscious experiences.

At present, two leading theoretical frameworks, Global Workspace Theory (GWT) and Integrated Information Theory (IIT), have emerged as prominent approaches, offering complementary perspectives on consciousness [66,82]. Another leading theory is the Dendritic Integration Theory (DIT) [83], which is based on physiological components and proposes that the state of consciousness arises at the level of deep pyramidal neurons, which are large excitatory neurons that hold a central position in both thalamocortical and corticocortical loops. Emerging evidence highlights the critical role of L5 pyramidal neurons in regulating conscious states [81]. These neurons, formerly described by Santiago Ramón y Cajal as “psychic cells”, integrate and coordinate cortical and thalamic processing [84]. According to Aru et al., L5 pyramidal neurons connect corticocortical and thalamocortical circuits, providing a bridge between the state and content of consciousness [83]. This unique role suggests that the L5 neurons are central to the mechanisms that regulate conscious awareness [83], as further confirmed by recent studies on anesthesia [85] reporting a decoupling between the basal and apical compartments of these neurons during the unconscious state, revealing a selective block of neural signaling in this condition. Notably, the same decoupling effect occurs with substances like ketamine, which inhibit NMDA glutamate receptors, thus suggesting a general mechanism underlying unconsciousness [83] which could be further analyzed through psychedelics.

Overall, psychedelics offer a powerful tool for exploring consciousness by inducing altered states of consciousness (ASC) [86,87]. In particular, classic serotonergic psychedelics profoundly affect sensory perception, mood, thought and the sense of self [88]. Among all altered states, NDEs and DMT-induced states stand out for their striking phenomenological overlap. In fact, NDEs consist of episodes of disconnected consciousness that typically occur in situations that involve an actual or potential physical threat (hypoxia, heart attack), or are perceived as such, and these experiences are characterized by a rich content with prototypical mystical features. Both involve a profound dissolution of the self, vivid encounters with hyper-real environments or entities, and a sense of timeless unity or transcendence, including space-time transcendence, experience of oneness and the perception of a hyper-dimensional reality [89]. From a biological perspective, a study published in Nature has found that there is a significant increase in endogenous DMT levels in the rat visual cortex following induction of experimental cardiac arrest [90], thus linking its release to NDEs, as most of them occur during cardiac arrests.

In conclusion, the combined study of L5 pyramidal neurons and psychedelic-induced altered states has advanced our understanding of the neural mechanisms underlying consciousness. L5 neurons appear to regulate conscious states through their role in synchronizing cortical activity, particularly under conditions such as anesthesia. At the same time, psychedelics allow researchers to probe disruptions in self-perception, cognition, and sensory integration, offering unique insights into altered and hybrid states of consciousness. By integrating these findings, neuroscience is beginning to bridge the gap between structural, functional, and phenomenological dimensions of consciousness, paving the way for a more unified and comprehensive understanding of this elusive phenomenon.

## 7. Conclusions and Future Perspectives

Psychedelics, including ayahuasca and its main component DMT, are receiving particular attention in the public opinion and medicinal field as breakthrough therapies for several psychiatric disorders, such as depression, anxiety, PTSD, and drug abuse. However, besides this comprehensible enthusiasm, transforming these therapeutics into the clinical routine requires more rigorous clinical trials in terms of controls with placebo, larger trial size and long-term studies.

Ayahuasca is lagging behind in terms of advancement of clinical studies compared to other psychedelics due to the lack of standard procedure for its preparation, composition and bioavailability. On the contrary, DMT and its analog 5-MeO-DMT are already undergoing phase 2 trials for TRD and MDD, through inhalation, intravenous, or intranasal administration, and the results are promising.

On this matter, it is also important to note that other psychedelics, such as MDMA and psilocybin, are at an advanced phase 3 stage of clinical development, with positive results in terms of efficacy and tolerability. This confrontation can be relevant in terms of financial investments and future studies, considering the costs and the challenges of marketing a new drug.

However, for advancing clinical research on psychedelics, there are several key aspects that need to be addressed with greater depth. First, it is essential to link the clinical efficacy of psychedelics to their molecular and cellular mechanisms, particularly at the level of the main brain networks. On this subject, advancements in neuroimaging and circuit-level analyses will offer new insights into their mechanisms of action.

Secondly, it is important to clarify the contribution of mystical and psychedelic experiences to the therapeutic outcomes, i.e., how these experiences play a pivotal role in mediating the psychological and emotional transformations observed. How is the psychedelics’ therapeutic efficacy associated with brain neuroplasticity and/or the mystical/spiritual experiences? And are they both equally needed? Currently, on this topic, there is no definitive consensus that subjective psychedelic experience is strictly necessary for therapeutic effects to occur. On one side, several studies have shown that intensity and qualitative features of the subjective experience, including mystical-type experiences, ego dissolution, emotional breakthrough, and psychological insight, are robustly correlated with positive clinical outcomes across conditions such as depression, anxiety, and substance use disorders [91]. On the other side, in recent years many non-hallucinogenic compounds have been produced to study separately neurobiological and hallucinogenic effects of psychedelics, such as tabernanthalog (TBG), a water-soluble, non-hallucinogenic, non-toxic analog of ibogaine [92]. In summary, current evidence supports a relevant association between the subjective psychedelic experience and therapeutic outcomes [93], yet it remains unresolved whether this relationship is causal, modulatory, or epiphenomenal.

So far, the only drug approved for TRD is esketamine, the (S)-enantiomer of ketamine, which promotes brain neuroplasticity at subanesthetic doses without generally inducing the whole psychedelic experience. Another issue that needs to be carefully addressed in the clinical efficacy of psychedelics is the challenge of identifying an appropriate placebo control group. The double-blind placebo control poses two problems. One is that patients might be aware if they are receiving active or placebo treatment, considering the relevance of the psychedelic experience. The second is that generally in psychedelic studies the placebo effect is particularly high, especially in patients with high expectations. Both aspects can generate bias in clinical results, and these aspects need to be considered. Different approaches have been proposed to reduce these biases like using active placebos, such as very low doses of psychedelics or another psychoactive compound [94,95]. Interestingly, in some studies that used a psychedelic-like setting, researchers found that more than half of the participants reported effects from an inactive substance, proving how the setting and expectations are powerful elements [96,97].

Finally, we should not underestimate the strong potential of psychedelics as tools to explore several aspects of human mind, including consciousness, NDE, and cognition, offering a powerful gateway to a deeper understanding of the human brain and its capacity for healing. Embracing this path still demands strict protocols and rigorous studies that can assure the safety of psychedelics, especially in the long term.

In this context, Carhart-Harris and Goodwin have presented a helpful framework to approach psychedelic promises and concerns, locating between cautious optimism—recognizing the real advances in this field—and constructive skepticism, pointing out the study limitations that need to be addressed in order to finally launch the new fascinating compounds into the market.

## Figures and Tables

**Table 1:** Major clinical trials investigating Ayahuasca, DMT, or its synthetic analogs in TRD (*Up to June 2025*).

Reference ID	Study Design	Enrollment	Date, Start—End	Aims	Arms and Interventions	Results
**NCT02914769** **(CNPq-466760/2014-0)** [68]	Phase 1/2—Interventional Study	*n* = 35	February 2014—December 2016	To test ayahuasca’s efficacy in TRD	**Experimental:** a single dose of ayahuasca **Placebo Comparator:** a single dose of a passive placebo	**HAM-D scores**: Significant improvement in patients treated with ayahuasca compared to placebo at day 7 (*p* = 0.019, *d* = 0.98) **MADRS scores**: Significant reduction in depression severity as early as day 1, with effects persisting through day 7. The difference between ayahuasca and placebo was significant at all time points: day 1 (*p* = 0.04, *d* = 0.84), day 2 (*p* = 0.04, *d* = 0.84), and day 7 (*p* < 0.0001, *d* = 1.49) **Response/remission rate**: At day 7, the response rate was 64% in the ayahuasca group versus 27% in the placebo group (*p* = 0.04), while the remission rate showed a trend toward significance (36% vs. 7%, *p* = 0.054) **Safety**: Well tolerated, with transient adverse effects; no serious adverse events
**NCT04698603** **(GH001-TRD-102)** [69]	Phase 1/2—Interventional Study	*n* = 16	November 2019—November 2021	To investigate safety, antidepressant effects, and dose-related psychoactive effects of inhaled GH001 (5-MeO-DMT) in TRD	**Experimental:** Phase 1 (Part A): GH001 dose A (12 mg) **Experimental:** Phase 1 (Part A): GH001 dose B (18 mg) **Experimental:** Phase 2 (Part B): GH001 Individualized Dosing Regimen (IDR) up to three increasing doses of 6 mg, 12 mg, and 18 mg	**MADRS scores**: Significant reduction from baseline at day 7: −21.0 (−65%) in the 12 mg group, −12.5 (−40%) in the 18 mg group, and −24.4 (−76%) in the IDR group (*p* < 0.0001) **Remission rate**: Remission (MADRS ≤ 10) at day 7 in 50% (12 mg), 25% (18 mg), and 87.5% (IDR) of patients (*p* < 0.0001). All remissions observed by day 1, 60% as early as 2 h after dosing **Response rate**: ≥50% reduction in MADRS from baseline in 75% (12 mg), 50% (18 mg), and 87.5% (IDR) patients **Safety**: Well tolerated, with mild/moderate transient adverse effects; no serious adverse events
**NCT05660642** **(BPL-003-204)** [72]	Phase 2a—Interventional Study	*n* = 12 (*Arm A*) *n* = 12 (*Arm B*)	February 2023—July 2025	To evaluate the safety, tolerability, and efficacy of intranasal BPL-003 (5-MeO-DMT) in patients with TRD	**Experimental:** In *Arm A* (on SSRIs-free), one of two doses of BPL-003 in Part 1, followed by two doses of BPL-003 in Part 2 **Experimental:** In *Arm B* (on SSRIs), one of two doses of BPL-003 in Part 1, followed by two doses of BPL-003 in Part 2	**MADRS scores**: in *Arm A* (on SSRIs-free), rapid antidepressant response (≥50% reduction in MADRS scores) observed in 55% of patients as early as day 2 (the day after dosing); in *Arm B* (on SSRIs), the mean MADRS score decreased by 18 points one day post-dosing (day 2), by 19 points after one month, and by 18 points after three months **Remission rate**: Sustained remission (MADRS ≤ 10) observed in 55% of patients on day 29 and in 45% of patients on day 85 **Safety**: Well tolerated in both arms, with no serious adverse events reported
**NCT05800860** **(GH001-TRD-201)** [70]	Phase 2b—Interventional Study	*n* = 81	May 2023—March 2025	To determine the safety and efficacy of inhaled GH001 (5-MeO-DMT) in TRD	**Experimental:** GH001 via inhalation as IDR with up to 3 escalating doses (6 mg, 12 mg, 18 mg) on a single day; subsequent doses given only if no peak experience at prior dose **Placebo Comparator:** Placebo via inhalation, IDR with the same dosing scheme as in the GH001 arm **Open-label extension (OLE):** up to 5 GH001 IDRs based on clinical response	**MADRS scores**: −15.2 (GH001) vs. +0.3 (placebo) at day 8; placebo-adjusted Δ = −15.5; *p < 0.0001* **Remission rate**: 57.5% of patients in the GH001 group achieved remission (MADRS ≤ 10) at day 8 vs. 0% in the placebo group (*p < 0.0001*); among OLE completers, 77.8% were in remission at 6 months; of those in remission at day 8, 91.7% maintained remission at 6 months **Secondary outcomes**: significant improvements in CGI-S, HAM-A, Q-LES-Q-SF compared to placebo (*p < 0.0001*) **Safety**: Well tolerated, with no evidence of treatment-emergent suicidal ideation or behavior; no serious adverse events
**NCT05870540** **(BPL-003-201)** [73]	Phase 2b—Interventional Study	*n* = 196	September 2023—June 2025	To evaluate the efficacy and safety of intranasal BPL-003 (5-MeO-DMT), with open-label extension, in patients with TRD	**Experimental:** Single low dose of BPL-003 **Experimental:** Single medium dose of BPL-003 **Experimental:** Single high dose of BPL-003 **Experimental:** Single mono-phasic dose of BPL-003 **Experimental:** Single biphasic dose of BPL-003 (administered as two nasal sprays minutes apart) **Placebo Comparator:** Single dose of active placebo	Results not yet available; publication expected by mid-2025
**NCT06094907** **(DMTPRD)** [74]	Phase 2—Interventional Study	*n* = 14	October 2023—March 2024	To evaluate the acute and subacute effects of inhaled DMT in patients with TRD	**Experimental**: administration of up to 2 inhaled doses of DMT within a single day (15 mg, followed by 60 mg) with a 1 h dose interval	**MADRS scores**: Significantly decreased from day 1, remaining significantly low up to one month after the dosing session: −24.33 on day 1, −22.0 on days 7, and 14, −17.0 in month 1 compared to baseline **PHQ-9 scores**: Decreases from baseline were observed on day 1 (*p* = 0.0008; *Hedge’s g* = 2.21), day 7 (*p* = 0.002; *Hedge’s g* = 1.69), day 14 (*p* = 0.003; *Hedge’s g* = 1.86), and after 1 month (*p* = 0.014; *Hedge’s g* = 1.22) **Response/remission rate**: Response of 83.3% and remission of 66.7% on day 7; response of 66.7% and remission of 50% after one month **Safety**: Well tolerated; mild/moderate adverse events on the day of administration

**Table 2:** Major clinical trials investigating ayahuasca, DMT, or its synthetic analogs in MDD (*Up to June 2025*).

Reference ID	Study Design	Enrollment	Date, Start—End	Aims	Arms and Interventions	Results
**NCT04673383 (CT026_001)** [76]	Phase 1/2a—Interventional Study	*n* (Part A) = 32 *n* (Part B) = 34	February 2021—December 2022	To evaluate the safety and tolerability of SPL026 (DMT fumarate) in healthy volunteers and the efficacy in MDD patients with moderate to severe depression	**Experimental:** ⊥ Healthy volunteers (Part A): SPL026 via IV injection ⊥ Patients with MDD (Part B): SPL026 via IV injection **Placebo Comparator:** ⊥ Healthy volunteers (Part A): SPL026-matched placebo to be administered by IV injection ⊥ Patients with MDD (Part B): SPL026-matched placebo to be administered by IV injection	**MADRS scores:** SPL026 reduced depressive symptoms, with a placebo-adjusted difference of −10.8 at week 1 (*p* = 0.002) and −7.4 at week 2 (−11.0 vs. −3.6; *p* = 0.02). At week 12, patients who received at least one active dose showed a mean MADRS reduction of −15.4 from baseline **Response/remission rate:** At week 1, 44% of patients achieved clinical response and 50% achieved remission (MADRS ≤ 10). At week 2, response and remission rates were 35% and 39%, respectively. Among those in remission within the first 3 months, 64% maintained remission at 6 months **STAI-T scores:** Significant improvements in anxiety symptoms, with a reduction of −13.5 vs. −1.9 at week 1 and −11.0 vs. −3.4 at week 2 compared to placebo **Safety**: Well tolerated; no serious adverse events reported; most adverse events were mild to moderate and resolved during the dosing session
**NCT05553691 (CT026_004)** [78]	Phase 1—Interventional Study	*n* = 17	December 2022—August 2023	To evaluate the safety, tolerability, and exploratory efficacy of a single dose of SPL026 (DMT fumarate) alone or in combination with SSRIs in patients with MDD	**Experimental:** Test cohort—single IV dose of SPL026 in patients on stable SSRI treatment with incomplete response **Experimental:** Control cohort—single IV dose of SPL026 in patients not receiving any pharmacological treatment for depression	**MADRS scores:** At week 4, mean MADRS dropped from 28.8 to 3.0 in the SSRI cohort and from 35.4 to 16.0 in the non-SSRI cohort **Response/remission rate:** Response (≥50% MADRS reduction) in 100% of SSRI patients vs. 80% in the non-SSRI group; remission (MADRS ≤ 10) was achieved by 92% vs. 20%, respectively **BDI scores:** Mean BDI decreased from 30.0 to 3.1 in the SSRI group, and from 36.0 to 14.8 in the non-SSRI group **STAI-T scores:** at 1 week, mean change was −22.2 with SPL026 (27.5 mg) + SSRI vs. −19.0 with SPL026 (27.5 mg) alone; at 2 weeks, −22.8 vs. −19.4, respectively **Safety**: Well tolerated in both cohorts; all adverse events were mild or moderate and transient; no serious adverse events reported; no increase in dissociation, suicidality, or psychosis

**Table 3:** Major clinical trials investigating ayahuasca, DMT, or its synthetic analogs in GAD (*Up to June 2025*).

Reference ID	Study Design	Enrollment	Date, Start—End	Aims	Arms and Interventions	Results
**NCT06051721** **(CYB004-002)** [80] **Status:** *recruiting*	Phase 2—Interventional Study	*n* = 36	May 2024—February 2025	To examine the safety, tolerability, pharmacokinetics, and preliminary clinical efficacy of CYB004 (deuterium analog of DMT) in moderate to severe GAD (GAD-7 score ≥10)	**Experimental**: two full IM doses of CYB004 administered three weeks apart, with EMBARK psychotherapy **Active Comparator**: two low IM doses of CYB004 administered three weeks apart, with EMBARK psychotherapy	Key safety and efficacy data were expected in early Q1 2025

## Data Availability

Not applicable.
